# One Health response to SARS-CoV-2-associated risk from mink farming in British Columbia, Canada, October 2020 to October 2021

**DOI:** 10.14745/ccdr.v48i06a04

**Published:** 2022-06-09

**Authors:** Veronic Clair, Elaine Chan, Adrianna Paiero, Erin Fraser, Rayna Gunvaldsen, Emily Newhouse

**Affiliations:** 1 British Columbia Centre for Disease Control, Vancouver, BC; 2 School of Population and Public Health, University of British Columbia, Vancouver, BC; 3 Department of Family Practice, University of British Columbia, Vancouver, BC; 4 Canadian Field Epidemiology Program, Centre for Emergency Preparedness and Response, Public Health Agency of Canada, Ottawa, ON; 5 Fraser Health Authority, Surrey, BC; 6 Ministry of Agriculture and Food, Victoria, BC

**Keywords:** SARS-CoV-2, COVID-19, mink farm, One Health, spillover, reservoir

## Abstract

**Background:**

Mink farms are susceptible to severe acute respiratory syndrome coronavirus 2 (SARS-CoV-2) outbreaks and carry an associated risk of novel SARS-CoV-2 variant emergence and non-human reservoir creation. In Denmark, control measures were insufficient to prevent onward transmission of a mink-associated variant, contributing to the nation-wide culling of farmed mink. To date, British Columbia (BC) is the only Canadian province to report mink farm SARS-CoV-2 outbreaks. The objective of this study is to describe BC’s One Health response to SARS-CoV-2-associated risk from mink farming, its outcomes, and insights from implementation.

**Methods:**

The detection of two mink farm outbreaks in December 2020 catalyzed BC’s risk mitigation response for both infected and uninfected farms, including the following: farm inspections and quarantines; Public Health Orders mandating mink mortality surveillance, enhanced personal protective equipment, biosafety measures and worker coronavirus disease 2019 vaccination, at-a-minimum weekly worker viral testing, and wildlife surveillance.

**Results:**

A One Health approach enabled a timely, evidence-informed and coordinated response as the situation evolved, including the use of various legislative powers, consistent messaging and combined human and mink phylogenetic analysis. Ongoing mink and worker surveillance detected asymptomatic/subclinical infections and facilitated rapid isolation/quarantine to minimize onward transmission. Voluntary testing and mandatory vaccination for workers were acceptable to industry; enhanced personal protective equipment requirements were challenging. Regular farm inspections helped to assess and improve compliance.

**Conclusion:**

British Columbia’s One Health response reduced the risk of additional outbreaks, viral evolution and reservoir development; however, a third outbreak was detected in May 2021 despite implemented measures, and long-term sustainability of interventions proved challenging for both industry and governmental agencies involved.

## Introduction

In 2020, Denmark reported community spread of a mink-associated severe acute respiratory syndrome coronavirus 2 (SARS-CoV-2) variant reducing antibody-mediated neutralization ((1,2)). Implemented measures, including surveillance, enhanced biosecurity and use of personal protective equipment (PPE), did not prevent SARS-CoV-2 transmission to other mink farms and humans ((3)), contributing to the Danish government’s decision to cull all farmed mink to prevent further mutation and spread ((3,4)).

Mink farm SARS-CoV-2 outbreaks had occurred in 12 countries ((5)) by the end of 2021, indicating high mink susceptibility ((6–10)). Mink-to-human transmission during mink farm outbreaks is, to date, the only confirmed animal-to-human SARS-CoV-2 transmission ((8,11,12)). The SARS-CoV-2 infection in a new non-human host is of public health (PH) concern because of viral adaptation to that host ((13–16)) and the potential creation of a reservoir. These factors can promote the emergence and re-introduction of variants of interest into humans ((13)) or other animals, further increasing opportunities for viral mutation ((14–18)).

Canadian ((19)), European ((15)) and international ((16)) organizations all recommend a One Health approach for managing SARS-CoV-2 risk on mink farms to enable a timely and coordinated response between the agricultural, animal health and human health sectors. The One Health approach also facilitates data sharing for surveillance and outbreak detection and response; however, there is a paucity of literature on the practical implementation, evolution and outcomes of such One Health approaches for mink farming ((14–16)). While the majority of Canadian mink farms are located in Eastern Canada, as of January 2022, British Columbia (BC) remains the only Canadian province with reported mink farm SARS-CoV-2 outbreaks ((5)). The objective of this work is to describe the implementation of BC’s One Health response to SARS-CoV-2-associated risk on mink farms from October 2020 to October 2021, detailing the interventions and outcomes, and discuss the insights gained.

## Setting

In 2020, all nine active mink farms in BC were located in the Fraser Health Authority (FH), near large urban centres. The mink fur industry was regulated and licensed by the BC Ministry of Agriculture, Food and Fisheries (MAFF), recently renamed to Ministry of Agriculture and Food. The industry operated in a cycle of breeding mink in spring, whelping offspring in summer and pelting in fall and winter, with pelts sold early the following year. British Columbia farms produced approximately 240,000 pelts in 2020 ((20)). Some farms operated independently, while two pairs of farms partially integrated operations, resulting in seven independent farm units.

Following the report of large SARS-CoV-2 outbreaks in mink farms in Europe ((5,15)), a provincial One Health Committee (OHC) was established in October 2020 to assess, mitigate and respond to risks from SARS-CoV-2 in farmed mink. Committee members included the BC Centre for Disease Control (BCCDC), FH, MAFF veterinarians and relevant organizations from other sectors, such as WorkSafeBC (**Table 1**). The OHC held weekly to semi-weekly meetings to share information and expertise, improve coordination and enable joint decision-making related to surveillance strategies, biosafety/control measures and other aspects of the One Health response (**Table 2**).

**Table 1:** Membership of British Columbia’s One Health Committee to address severe acute respiratory syndrome coronavirus 2 risk on British Columbia mink farms

Organization	Role/mandate
British Columbia Centre for Disease Control	Providing provincial public health leadership in British Columbia and acting as Chair
Fraser Health Authority	Regional health authority with jurisdiction for local outbreak management under the *BC Public Health Act*
WorkSafeBC	Overseeing the protection of workers, including mink farmworkers
Ministry of Agriculture, Food and Fisheries	Regulatory responsibility of fur farming, animal health programs, and control of reportable animal diseases
Ministry of Forests, Lands, Natural Resource Operations and Rural Development	Responsible for wildlife monitoring and issuing export permits for mink pelts
Ministry of Environment	Regulatory responsibility for environmental discharge (as needed)
Canadian Food Inspection Agency	Providing technical expertise (as needed)

**Table 2:** Sequential interventions to manage and mitigate risk from severe acute respiratory syndrome coronavirus 2 on mink farms in British Columbia, October 2020 to October 2021

Trigger	Purpose and considerations/actions	Outcomes and challenges
1. OHC, formed in October 2020		
Large outbreaks reported in mink farms in European countries	Purpose: To assess, mitigate, and respond to risks from SARS-CoV-2 on BC mink farms using a One Health approach. Action: The OHC held weekly to semi-weekly meetings to: share information and contextualized technical and on-the-ground expertise of all members; coordinate human, animal, and environmental surveillance strategies; jointly identify biosafety gaps to be addressed and request for funds or other response tools; collaborate on decision-making based on shared situational assessments and evidence review; coordinate communication with mink farm operators; and liaise with other jurisdictions and organizations such as the Public Health Agency of Canada, United States Centers for Disease Control and Prevention and the World Health Organization.	The OHC enabled timely and effective response to mink farm outbreaks, realistic and implementable regulations, policies and guidelines, and optimization and sharing of technical, financial and human resources. The OHC also helped to provide unified and coordinated messaging to mink farm operators. Challenges in differing perceptions of risk or related decisions were typically surmountable and consensus was possible to reach in most areas. In some circumstances where specific legal jurisdiction clearly identified the most responsible organization, decisions were left to that organization.
2. Mink farm inspections, starting December 4, 2020		
2.1 Initial inspections at Farm 1		
Outbreak investigation at Farm 1	Purpose: To assess adherence to enhanced biosafety measures and identify gaps for improvement. Action: Coordinated inspections were carried out by OHC partners (i.e. PH, MAFF, and/or WorkSafeBC).	Inspections at Farm 1 found limited biosafety measure implementation. Out of concern for workers potentially contracting a mink-adapted COVID-19 variant, only activities necessary for animal welfare were immediately allowed to continue at Farm 1, halting the pelting process. Based on Farm 1 findings, a letter was issued to all mink producers urging the implementation of enhanced biosafety measures as outlined by the draft federal guidelines.
2.2 Repeated farm inspections on all mink farms		
Finding of limited safety measures at Farm 1	Purpose: To monitor implementation and feasibility of biosafety measures required by PH, MAFF or WorkSafeBC. Action: Inspections were repeated at all active mink farms on an ongoing basis.	Initial inspections on all mink farms found weaker biosafety measure implementation than those recommended by the mink farm biosafety advisory group. Implementation of recommended enhanced biosafety measures improved over time with the issuance of a FH PH Class Order mandating enhanced measures, along with subsequent inspections and feedback to mink farm operators.
3. Formal communications with mink farm operators, including Provincial Health Officer and Chief Veterinarian letter to operators on December 6, 2020, and follow-up meetings between PH, MAFF and industry in January and February 2021		
Weak biosafety measures observed on Farm 1 during outbreak investigation, and in other farms’ inspections triggered by the Farm 1 outbreak	Purpose: To communicate PH concerns to mink farm operators and achieve improved biosafety measures on mink farms. Action: The letter reminded operators of the mandatory requirement for a written COVID-19 Safety Plan and for those plans to be posted. It strongly recommended all mink farms to immediately review and strengthen those Safety Plans to implement the measures recommended for mink farms that were outlined in a biosecurity advisory from the Canadian Food Inspection Agency and the Public Health Agency of Canada. Those measures included the use of fitted respirators (N95 or equivalent) especially for pelting (or, if unavailable, medical masks), gloves, and eye protection, as well as viral testing of workers before pelting and on a weekly basis until pelting conclusion. Follow-up meetings (one with a “town hall” format) were instituted for mink farm operators to share information about the industry operations, for public health and MAFF to share more about the science, and to support discussion about control measures.	Some operators’ COVID-19 Safety Plans were found lacking, and some reported that they thought recommendations were challenging, confusing, and unnecessary. To improve compliance, FH issued a Class Order to all mink farms mandating enhanced measures be implemented before pelts, animals, or products could be moved on or off farms. OHC subcommittees were also created to issue BC-specific biosafety recommendations balancing risk reduction with practicality considerations and challenges. Meetings led to greater understanding of mink farm operations and increased overall buy-in for public health measures, although perceptions still varied across the industry.
4. Mink euthanasia for the purpose of pelt production on Farm 1, December 16–24, 2020		
Concern that maintaining a stock of thousands of mink infected with SARS-CoV-2 at Farm 1 would promote further viral replication and mutation, on the one hand, versus concern of viral transmission to workers	Purpose: To decrease further viral replication with associated risk of mutation among infected mink on Farm 1. Considerations: There were thousands of animals left to skin at Farm 1 when the process was halted. On the one hand, keeping the herd at its large size could enable further viral replication and promote the emergence of more mutations, and from the producer’s perspective, mink needed to be skinned as soon as possible before aging decreased pelt value, among other considerations. On the other hand, the skinning process is considered high risk because of compression of the mink’s lungs expelling respiratory secretions, potentially generating aerosols, and workers being in very close proximity to each other and to the mink. Culling of the entire herd, disposal, and disinfection was considered, to decrease ongoing risk of transmission from regular operation. However, this was ultimately decided against as it would have exposed a significant number of additional workers, was logistically complicated and had significant negative implications for the producers.	A decision was reached to allow euthanasia and skinning under strict biosafety measures, which could vary considering whether performed by previously infected workers or not. Farm 1 producer decided to proceed with euthanasia/skinning. Skins were not ultimately processed into pelts as processing facilities were unable to accept skins from an infected herd, causing financial strain.
5. Surveillance of farmed mink mortalities, starting in December 2020		
Concerns of potentially undetected or delayed detection of mink outbreaks	Purpose: To quickly detect SARS-CoV-2 infection in mink herds. Considerations and action: The OHC had concerns that clinical surveillance with weekly written monitoring of illness and mortality, as suggested by the Canadian COVID-19 One Health working group ((21)) was unlikely to be adequate, and active surveillance had been recommended by both the World Health Organization and the World Organisation for Animal Health ((16)). Farm 1 had submitted mortalities upon request by MAFF following detection of the farmworker outbreak, while Farm 2 submitted mortalities for testing based on herd signs or excess mortality. Participation in mandatory mink mortality surveillance regardless of excess mortality or compatible signs was ordered December 14, 2020, with the start of collection in the following month. The goal of mandatory mortality surveillance was to monitor for SARS-CoV-2 infection among mink herds in a timely manner, regardless of signs or symptoms, enabling swift quarantine and detection of mutations and minimizing transmission to workers. Based on the Canadian Food Inspection Agency guidelines, it was estimated that weekly collection of 15 mink mortalities per farm would provide a 95% surveillance sensitivity to detect an outbreak; therefore, farms were required to provide up to 15 per week. Logistical considerations, both from farms and from testing processing capacity, suggested five mortalities per week would be more feasible, estimated to provide 65% sensitivity. FH environmental health officers collected frozen and sealed mink carcasses from both non-infected and infected premises each week and brought them to MAFF’s Animal Health Centre for SARS-CoV-2 testing. Any non-negative samples were sent to the National Centre for Foreign Animal Disease for confirmatory polymerase chain reaction testing and to the BCCDC Public Health Laboratory for whole-genome sequencing, if positive.	On December 23, 2020, mink mortalities collected the prior week from a second farm (Farm 2) returned SARS-CoV-2-positive, and an additional outbreak was declared; mink displayed slight clinical signs and increased mortality (fewer than 3%). Farm 2 owners euthanized their small herd (fewer than 1,000 mink) without request by PH or MAFF. Farms had challenges in providing even the five mortalities per week due to the low mortality rate for many months of the year and small herd sizes in BC. On May 14, 2021, Farm 3 mink mortalities collected in early May were confirmed SARS-CoV-2-positive. The outbreak investigation uncovered mink exposure to an infectious worker who tested positive approximately 6 weeks earlier (within 14 days of the first dose of the vaccine), harbouring the same strain as positive mink. Ongoing mortality collection enabled detection of Farm 3 mink cases months after the outbreak start, enabling timely assessment of viral propagation and evolution in the herd; however, mink mortality freezing, collection, thawing and testing was resource-intensive.
6. Farm quarantine by BC Chief Veterinarian		
Suspicion or confirmation of a SARS-CoV-2 infection in a mink herd (Farm 1, Farm 2 and Farm 3)	Purpose: To limit potential spread of the virus from infected mink farms. Action: The Chief Veterinarian placed a quarantine order on infected premises that restricted all movements of animals, products and goods off of the farm. New enhanced protocols of disinfection of vehicles, products, and goods were put in place before authorization was given for non-essential activities.	On Farm 1, the herd was deemed free of disease as of February 24, 2021, after 2 sets of 65 samples taken 2 weeks apart were found to be all negative. As the Farm 2 herd was culled, it did not need to be declared free of disease. The Farm 3 herd was still considered infected by the end of this study period. Farm sites remained in quarantine until the determination that the farm environment was decontaminated.
7. Mandatory worker COVID-19 testing, December 2020–January 2021		
Concerns of undetected asymptomatic worker infection or avoidance of testing by symptomatic workers	Purpose: To detect past or current COVID-19 infection in mink farmworkers. Action: In Mid-December 2020, mink farmworkers (n=102) were mandated to complete COVID-19 virology and serology tests before being allowed back on farms. Farm 2 workers had repeated viral and serological testing in January 2021 after pelting completion, to detect missed infections.	No viral or serological tests taken in December 2020 and January 2021 returned SARS-CoV-2-positive.
8. Voluntary worker COVID-19 surveillance, starting in January 2021		
Concerns of undetected asymptomatic worker infection or avoidance of testing by symptomatic workers	Purpose: To improve detection of COVID-19 infection in mink farmworkers. Action: Public Health implemented a free-of-charge, weekly surveillance program for mink farmworkers in January 2021, utilizing self-collected saline gargle samples ((22,23)). BCCDC PH nurses trained workers on gargle sample self-collection and associated processes, minimizing ongoing PH staffing requirements and increasing testing acceptability, while maintaining sensitivity compared to nasopharyngeal swabs ((22,23)) and still enabling whole genome sequencing by the BCCDC Public Health Laboratory. A same-day medical courier collected samples from farms for delivery to the BCCDC Public Health Laboratory, with 0–2 days from sample collection to results. Indeterminate results led to repeated tests.	All active farms (6 farm units including Farm 1) participated by the end of February 2021. Between February 21 and May 31, 2021, an audit showed active workers’ weekly participation at 86%–100% per farm. The worker surveillance program detected 11 COVID-19 cases. One additional positive worker was detected through community testing following household exposure. Detection of positive workers triggered increased testing (2–3 times per week). Further, if an infectious worker had been near mink, 3 weeks of live mink sampling also occurred. Some farms voluntarily maintained twice or thrice-weekly testing.
9. Wildlife surveillance, starting in January 2021		
Concern regarding SARS-CoV-2 transmission to surrounding wildlife	Purpose: To detect potential SARS-CoV-2 transmission to wildlife from escaped mink or feral cats ((17,18,24)). Action: Wildlife surveillance around Farm 1 and Farm 2, utilizing wildlife trapping, testing, and video footage, occurred from January to March 2021. Wildlife surveillance was also undertaken around Farm 3 in summer 2021.	Virology and serology tests were negative on all 65 animals sampled around Farm 1 and Farm 2. Repeated wildlife surveillance surrounding Farm 3 in summer 2021 located no infected wildlife but did detect 3 escaped mink that tested positive ((25)).
10. Mandatory worker COVID-19 vaccination, April 2021		
Availability of COVID-19 vaccine supply and prioritization of vaccines for high-risk workplaces, including mink farms	Purpose: To reduce risk of SARS-CoV-2 transmission to mink herds from mink farmworkers. Action: On April 15, 2021, a new FH PH Order permitted only vaccinated workers to work in proximity to mink.	Most workers opted to be vaccinated (~90% first dose at the time of the Order including unmandated workers). Pfizer-BioNTech (BNT162b2) vaccination was offered to workers beginning March 17 and subsequently to their household members with excellent uptake; second doses were offered in May–June, with more than 90% worker uptake. Out of the 12 COVID-19-positive workers, 33% were unvaccinated, 25% partially vaccinated (onset or positive test more than 14 days post-first dose), and 42% fully vaccinated (more than 14 days post-second dose) at the time of infection.
11. Joint rapid qualitative risk assessment, June 2021		
Need for an up-to-date, BC-specific assessment on the risk of mink farm-related SARS-CoV-2 variant of interest emergence and community transmission to inform further response	Purpose: A formal risk assessment was undertaken to support decision-making regarding concerns related to SARS-CoV-2 and the mink farm industry in BC. Action: In June 2021, a multi-jurisdictional risk assessment was conducted as per best practices ((26)). National and provincial experts assessed potential scenarios’ probabilities, impacts, and uncertainties, using a modified Delphi approach (*personal communication, V. Clair, 2021*).	The likelihood of a variant of interest emerging in mink and circulating in the community over the next 5 years was evaluated as unlikely (moderate-high uncertainty) with minor to moderate impacts (moderate-high uncertainty). As a result, BCCDC recommended a moratorium on mink farming expansion. Following detection of SARS-CoV-2-positive escaped mink on Farm 3, the Provincial Health Officer issued a moratorium on expansion of the mink industry in late July 2021 ((27)).

Abbreviations: BC, British Columbia; BCCDC, British Columbia Centres for Disease Control; COVID-19, coronavirus disease 2019; FH, Fraser Health Authority; MAFF, Ministry of Fisheries and Oceans; OHC, One Health Committee; PH, Public Health; SARS-CoV-2, severe acute respiratory syndrome coronavirus 2

Under the BC Animal Health Act ((28)), SARS-CoV-2 infection in animals was made reportable; mink farms and herd veterinarians were to report mink signs or symptoms compatible with SARS-CoV-2, including excess mortality, as well as any confirmed infections. By November 2020, MAFF had informed all mink farms of SARS-CoV-2-associated risks and assessed biosafety measures. Mink farm operators reported implementing physical distancing, signage related to not working while sick and non-medical mask use. A set of draft federal guidelines ((19)), shared with the BC Mink Producers Association by the OHC, recommended implementation of further biosecurity measures. In November 2020, PH attempted to discuss enhanced measures with a tepid response by industry.

On December 2, 2020, a SARS-CoV-2 outbreak was detected on mink Farm 1 ((29)), triggering urgent OHC meetings to optimize outbreak management and a coordinated provincial response. The Farm 1 outbreak ultimately involved 11 coronavirus disease 2019 (COVID-19) cases among 12 workers ((29)). Mink on Farm 1 displayed few clinical signs and fewer than 1.5% mortality.

## Interventions, challenges and outcomes

**Figure 1:**
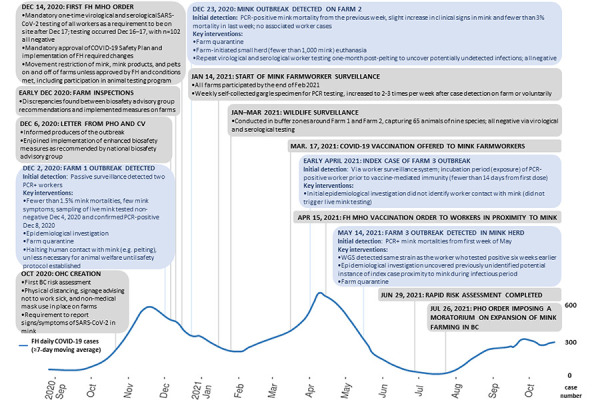
Timeline of significant events and interventions in public health response related to severe acute respiratory syndrome coronavirus 2 in mink production in British Columbia, Canada, 2020 to 2021 Abbreviations: BC, British Columbia; COVID-19, coronavirus disease 2019; CV, Chief Veterinarian; FH, Fraser Health Authority; MHO, Medical Health Officer; OHC, One Health Committee; PCR, polymerase chain reaction; PHO, Provincial Health Officer; SARS-CoV-2, severe acute respiratory syndrome coronavirus 2

The One Health approach included on-going evidence review and risk assessment informing the response. The main actions implemented were farm inspections, the use of public health orders to mandate worker testing and vaccination, mink mortality viral surveillance and biosafety control measures, a voluntary asymptomatic worker viral surveillance system, and wildlife surveillance. Table 2 and **Figure 1** provide a timeline and full details on the triggering events, actions, challenges and outcomes of BC’s One Health response. Other measures on infected farms included animal and human epidemiological investigations, live animal testing, biocontainment and disinfection measures and quarantine of sites and workers ((29)).

Initial farm inspections revealed weak biosafety measure implementation, resulting first in communication encouraging strengthening of those measures followed by a Public Health Order mandating specific measures (**Table 3**). Before implementation of improved biosafety measures and availability of human vaccine, an outbreak in mink on a second farm (Farm 2) was detected due to the herd displaying slight clinical signs and increased mortality (fewer than 3%). The Farm 2 owners euthanized their small herd (fewer than 1,000 mink) without request by PH or MAFF. After the Farm 1 outbreak, all mink farmworkers in FH (n=102) were mandated to complete COVID-19 viral and serological testing, with no infections detected. After the Farm 2 outbreak, Farm 2 workers underwent a second round of viral and serological testing, again with no worker infections detected.

**Table 3:** Public Health Orders in British Columbia relating to mink farms, 2020 to 2021

Public Health Orders	Descriptions
December 14, 2020: Fraser Health Authority Medical Health Officer Order	
COVID-19 Safety Plans and enhanced biosecurity	Mandating provision of the COVID-19 Safety Plans to FH, for review and approval by FH. As part of the safety plan requirements, enhanced use of personal protective equipment was required, including the usage of N95 or equivalent protection, eye protection, protective clothing, safety footwear that can withstand disinfection, for all activities occurring in close proximity to mink or mink feed.
Worker registry and human testing	Providing a list and contact information of all employees, contractors, volunteers, owners and operators or other individuals who have worked at the mink farm in the last three months, to facilitate epidemiological investigation if needed and ascertain workers compliance with testing or other measures as needed. Asymptomatic testing of all mink farmworkers (not already tested) to occur by a specific date, after which untested workers would not be allowed on premises, for initial case ascertainment. Serological testing of workers, to clarify if workers might have had an infection in the past.
Animal surveillance and testing	Mandating participation in an animal surveillance system, to be specified by FH, which included weekly submission of mink mortality for testing, with the hope of detecting infections early in asymptomatic herds to implement further monitoring and risk mitigation measures to prevent mink related strain transmission and spread to humans.
Movement restriction of mink, mink products, and pelt	Restriction on moving mink or mink-related products between farms, to limit opportunities for the spread of the virus, which occurred in other zoonoses outbreak in BC and COVID-19 outbreaks in other countries. Restriction on moving pelts until compliance with the terms of the order, as assessed by FH.
April 15, 2021: Fraser Health Authority Medical Health Officer Order	
Vaccination	Mandatory vaccination of workers who work in close proximity to mink. Mandatory record keeping of workers’ vaccination status.
July 26, 2021: Provincial Health Officer Order	
Moratorium on mink farming expansion	Farms must report the number of breeding mink stock, non-breeding mink and total mink on the farm. Must not allow the number of breeding mink or non-breeding mink stock to exceed their reciprocal number as the date of this order. Must not acquire new live mink.

Abbreviations: BC, British Columbia; COVID-19, coronavirus disease 2019; FH, Fraser Health Authority

Mandatory vaccination and the worker COVID-19 surveillance program were acceptable to the industry; however, mandatory enhanced PPE usage and other biosafety measures were challenging. Skepticism regarding effectiveness or necessity, costs and discomfort of PPE constituted some of the impediments. To address challenges with specificity and feasibility of national biosafety recommendations, a local OHC subcommittee was formed to swiftly generate BC-specific recommendations. An effective short-term mode of compliance was the restriction of pelt, animal and product movement unless biosafety requirements were met. Ongoing farm inspections were also helpful in assessing and improving compliance.

**Figure 2:**
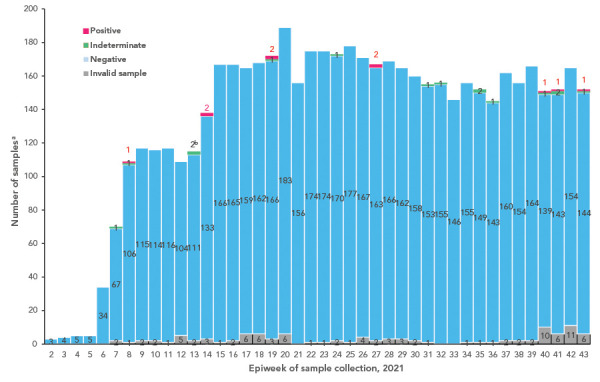
Mink farmworker coronavirus disease 2019 surveillance virologic testing results, January 14 to October 31, 2021 ^a^ The number of weekly samples exceeds the number of mink farmworkers as sampling was increased to twice or three times weekly after detection of a case on some farms; the number of workers per farm varies significantly depending on the mink farm production cycle phase. As of October 31, 2021, 11 cases of coronavirus disease 2019 infection in mink farmworkers were detected through the human surveillance program (10 positive via saline gargle and 1 indeterminate on gargle subsequently positive on follow-up nasopharyngeal [NP] swab) out of 5,673 tests among 123 unique workers since January 14, 2021 ^b^ One individual who returned an indeterminate result during epiweek 13 was positive on follow-up NP swab

After implementation of strengthened biosafety measures, mink mortality surveillance and voluntary worker surveillance, only small clusters of human cases (one or two persons/cluster) occurred between January 14 and May 31, 2021, unlike the outbreak among workers on Farm 1 in December 2020 ((29)) prior to enhanced measures (**Table 4** and **Figure 2** detail findings from worker surveillance).

**Table 4:** Coronavirus disease 2019-positive mink farmworkers in British Columbia, January 14 to October 31, 2021

Date range	N	%	Context
January 14 to March 17, 2021	2^a^	16.6	Before vaccination being offered to farmworkers
March 18 to May 31, 2021	5	41.6	After offering vaccination (mandatory vaccination not in place until April 15, 2021): n=1 had chosen not to be vaccinated n=1 was positive within 14 days of vaccination with first dose (not considered partially immunized) n=3 were positive more than 14 days after receipt of a first dose of vaccine (considered partially immunized)
June 1 to October 31, 2021	5	41.6	Following receipt of 2 doses of vaccine: n=5 were considered fully vaccinated and part of the outbreak on Farm 3
Total	12	100.0	Human cases arose on 3 of 6 remaining farm units^b^ from the start of the surveillance system until the end of the study period

^a^ One worker was detected through community testing following household exposure rather than through the worker surveillance system

^b^ Of the original nine active mink farms in British Columbia, two pairs of farms were submitting human surveillance samples jointly as they had integrated operations and were in close proximity to each other with workers working on both sites. It was not possible to separate these farms through the human surveillance system. From these original seven independent farm units, one farm (Farm 2) had ceased operation, culling all of its mink after an outbreak was detected in the mink herd, resulting in six remaining farm units by January 2021

One worker case triggered a mink herd outbreak on a third farm (Farm 3). Farm 3 was the only outbreak detected through the mink mortality testing, with phylogenetic analysis identifying the same strain as a previously positive worker who had not originally been thought to have had contact with mink. Mortality surveillance enabled timely monitoring of SARS-CoV-2 propagation and evolution in Farm 3 over several months, despite a lack of symptoms of infection ((29)). Following two-dose vaccination of workers in contact with mink, with more than 90% uptake among all workers, there were no further mink herd outbreaks, although five worker cases were detected through the surveillance system during this time.

Wildlife surveillance around Farm 1 and Farm 2 occurred in January to March 2021, to detect potential SARS-CoV-2 transmission to wildlife from escaped mink or feral cats. Virology and serology tests were negative on all 65 animals sampled ((25)). Repeated wildlife surveillance surrounding Farm 3 in summer 2021 also failed to detect infected wildlife but located three escaped mink that tested positive.

In June 2021, a formal risk assessment was conducted based on best practices (**Table 5**). After consideration of the risk portrayed in the report, BC’s Provincial Health Officer placed a moratorium on mink farming expansion in the province ((27)).

**Table 5:** Joint rapid qualitative risk assessment on severe acute respiratory syndrome coronavirus 2-associated risk for mink farms in British Columbia, June 29, 2021

Methods
The scope of the assessment was limited to the mink farms in the Fraser Health Authority and British Columbia. The outcome of interest was the circulation of a mink-induced SARS-CoV-2 variant of interest (VOI) in the community that could potentially increase transmission, cause more severe disease in humans, escape vaccines or significantly decrease the effectiveness of therapeutic and diagnostic technologies, compared with what is seen with currently circulating variants. The pathways assessed were human-mink-human and human-mink-wildlife-human. Two timeframes were assessed: 1) short term: completion of the current production cycle, up to before the start of the next breeding season; and 2) long term: the next five years. A multi-jurisdictional expert group jointly completed all steps of the process. Using a modified Delphi approach, the expert group assessed probability, impact, and uncertainty estimates. Probabilities along the scenario pathways were combined as per accepted qualitative risk assessment methodologies. Several assumptions were made by the expert group that influence the results. There is often a high degree of uncertainty related to the assumptions. They are important to highlight because changes to the assumptions may affect the final estimates, and significant changes may indicate the need for reassessment.
Probability level, impact and uncertainties
The combined assessment of probability and the assessment of consequence the emergence and circulation of a VOI in the community of mink/wildlife origin were as follows: 1. What is the likelihood and impact of emergence and circulation of a SARS-CoV-2 VOI in the community due to virus evolution in mink or “wildlife after exposure to mink” during completion of **this cycle**, compared to what is seen with currently circulating variants and evolving public health measures? **Probability:** very unlikely (VOI in wildlife pathway) to very unlikely/unlikely (VOI in mink pathway) **Uncertainty:** moderate (VOI in mink pathway) to high (VOI in wildlife pathway) **Impact:** minor to moderate at the local/regional level, and slightly less at the provincial level **Uncertainty:** moderate to high 2. What is the likelihood and impact of emergence and circulation of a SARS-CoV-2 VOI in the community due to virus evolution in mink or “wildlife after exposure to mink” **WITHIN THE NEXT FIVE YEARS**, compared to what is seen with currently circulating variants and evolving public health measures? **Probability:** very unlikely (VOI in wildlife pathway) to unlikely (VOI in mink pathway) **Uncertainty:** moderate (VOI in mink pathway) to high (VOI in wildlife pathway) **Impact:** minor to moderate at the local/regional level, and slightly less at the provincial level **Uncertainty:** moderate to high The combined probability estimates for both timeframes of the human-mink-human pathway were driven primarily by the probability of the evolution of the virus into a VOI in a mink herd, with higher uncertainty associated with the probability in the five-year assessment due to the higher uncertainty in the expected number of mink herd outbreaks per year as time goes on and higher uncertainty regarding the evolution of a VOI. The risk assessment for the next five years assumed limited control measures. In the pathway involving wildlife, most steps were estimated as less probable than for the direct pathway from mink-to-humans, with a similar level of uncertainty. The mode of the overall probability for the human-mink-wildlife-human pathway during both periods was very low, regardless of the spread scenario in wildlife (limited spread or reservoir). These estimates were driven primarily by 1): the probability of evolution of the virus into a VOI in wildlife that was assessed as very unlikely to occur and 2) the probability that a person would contract the virus from wildlife was assessed as very unlikely. Experts expressed it is more likely that a VOI will arise in humans rather than in mink. If there was emergence and circulation of a VOI in the community that was of mink/wildlife origin, the magnitude of the impact on the health of the population above the current/ongoing pandemic impacts for this cycle were estimated as likely to be minor to moderate at the local/regional level, and slightly less at the provincial level. The uncertainty associated with this was moderate to high. The magnitude of the impact at the five-year timeframe was assessed as likely to be similar, with a higher level of uncertainty.

Abbreviations: SARS-CoV-2, severe acute respiratory syndrome coronavirus 2; VOI, variant of interest

## Discussion

The OHC realized all the benefits of a timely, coordinated, evidence-based and jointly accountable One Health response ((30)). The main interventions, which were similar to responses in other jurisdictions ((3,6)), included sequential situational assessments followed by voluntary and mandated measures such as human, mink and wildlife surveillance, farm inspections, enhanced biosafety measures and a moratorium on mink farming expansion. While we did not find evidence of spread between farms or to the community following implementation of interventions akin to Denmark ((3,4)), phylogenetic analyses indicated mink-to-human transmission at the subsequent Farm 3 outbreak despite enhanced biosafety measures and two-dose worker vaccination ((29)).

### Strengths and limitations

Existing PH and animal health regulations were paramount in improving compliance with new interventions and/or measures. The sequential approach enabled the response to continually adapt as the situation evolved, considering new scientific evidence and past successes, challenges, and outcomes. Regarding OHC’s joint decision-making, consensus on most approaches was reached in a timely manner because of the ongoing dialogue and sharing of information. Some decisions clearly lay within a single organization’s purview and consensus was not required; however, the One Health approach enabled effective coordination and integration of multiple perspectives into decision-making.

The first two mink farm outbreaks occurred in December 2020 during the second COVID-19 wave in BC, before vaccination, when minimal biosafety measures were in place, and with increased staffing and mink-worker and/or worker-worker interactions during the pelting season. Structural disincentives for testing among farmworkers ((31–33)) may have delayed worker testing at Farm 1, thus delaying outbreak detection ((29)). Combined, ongoing human and mink mortality surveillance were able to successfully overcome case detection difficulties such as asymptomatic/mildly symptomatic mink and human infection ((34,35)) or testing avoidance ((31–33)). Rapid human and mink case detection from surveillance also enabled timely whole genome sequencing and combined phylogenetic analyses.

Only one outbreak occurred following implementation of interventions, despite human cases detected on three out of six farm units in January–October 2021, suggesting that our multi-layered approach including PPE, biosafety measures, surveillance and mandatory worker vaccination combined to reduce outbreak risk. A systematic review indicated that physical distancing of more than one metre substantially decreased human-to-human transmission (adjusted odds ratio [AOR] 0.18, 95% CI 0.09–0.38), as did consistent use of face masks (AOR 0.15, 95% CI 0.07–0.34), with stronger associations with respirators ((36)). Lapses in PPE usage and other biosafety measures would be unsurprising, as these occur even in healthcare settings where workers receive extensive training and monitoring ((37–39)). The human surveillance program also decreased the probability of a worker COVID-19 outbreak occurring, further decreasing the risk of transmission to the herd. In humans, modelling studies suggest weekly testing reduces secondary infection by 23%–60%, increasing to 90% with twice-weekly testing ((40,41)). As no outbreaks were seeded after workers had spent 14 days following dose one vaccination, immunization with a highly effective vaccine likely further decreased outbreak risk over the next five months, past the peak of the fourth wave in BC. Despite support from the literature with plausible timelines and mechanisms suggesting the control measures were effective to a point, it is difficult to establish causality between measures and the number of cases or outbreaks detected after their implementation.

Without mandatory mink mortality surveillance, the outbreak on Farm 3 might not have been detected until much later, if at all, in part because SARS-CoV-2 infection in mink frequently results in asymptomatic or mildly symptomatic infections ((42)). The BC farms had difficulty providing even five mink mortalities weekly, lowering the estimated infection detection sensitivity to less than 65% ((21)). With at least weekly worker surveillance and infectious worker contact with mink triggering live mink testing as part of our One Health approach, it is unlikely an outbreak was missed.

Spillover into wildlife from infected mink herds and associated feral cats could promote the emergence of SARS-CoV-2 genetic mutation of concern or of a reservoir ((17,18,24)). Repeated wildlife surveillance surrounding all three infected farms failed to detect infected wildlife, despite locating three escaped mink that tested positive. A limitation of this monitoring was that the sensitivity of the wildlife surveillance was uncertain ((43,44)).

One of the main limitations of BC’s comprehensive One Health response was its resource-intensive nature. Evidence review, risk assessment, mink mortality surveillance and inspections all were resource-intensive at a time when most of the OHC’s organizations were already overstretched by the pandemic response. Although the use of self-collected saline gargle specimens for human surveillance decreased PH resource requirements while maintaining sensitivity and improving acceptability ((22,23)), the materials needed, specimen transportation, and ongoing lab processing and analysis were not without cost.

### Implications

In BC, sustaining many of the implemented interventions long term, despite some evidence of their effectiveness, was challenging for the industry and the various agencies involved. Worker vaccination likely reduces the risk of subsequent outbreaks and is less resource-intensive, but is contingent on vaccine effectiveness against prevailing strains, which continues to evolve. Furthermore, immunization does not resolve infection detection difficulties in workers and mink. Without ongoing worker and mink herd surveillance, it is possible that mink farm outbreaks and the associated risk of mink-related viral adaptation and transmission back to the community are occurring undetected in other jurisdictions including other provinces.

## Conclusion

A One Health response tailored to mitigating SARS-CoV-2 risk on mink farms in BC, led by an issue-specific OHC, was triggered following two mink farm outbreaks in December 2020. The One Health approach enabled ongoing communication between relevant agencies and a timely and coordinated response. A third mink farm outbreak occurred in mid-2021 despite implemented enhanced PPE and biosafety measures, worker and mink surveillance programs and regular farm inspections. A comprehensive One Health approach, involving animal health, public health, worker safety and industry regulation organizations, should be implemented to respond to complex and evolving threats such as risks from emerging zoonotic pathogens in farmed animals.
